# Association between alcohol consumption and latent fasting blood glucose trajectories among midlife women

**DOI:** 10.3389/fpubh.2024.1331954

**Published:** 2024-01-24

**Authors:** Xingzhou Wang, Song Lin, Xiwei Wang, Pengxia Gao, Juan Chen

**Affiliations:** ^1^Department of Endocrinology, The Affiliated Huaian No.1 People’s Hospital of Nanjing Medical University, Huai’an, Jiangsu, China; ^2^Department of Clinical Nutrition, The Affiliated Huaian No.1 People’s Hospital of Nanjing Medical University, Huaian, Jiangsu, China; ^3^Department of Mathmatics, University of Liverpool, Liverpool, United Kingdom

**Keywords:** alcohol, trajectory, blood glucose, sex hormones, midlife women

## Abstract

**Background:**

This investigation sought to elucidate the correlations between alcohol intake and trajectories of fasting blood glucose (FBG) among American women in midlife.

**Methods:**

Our analysis was rooted in the foundational data from the Study of Women’s Health Across the Nation (SWAN), a comprehensive longitudinal study centered on US women during their midlife transition. We employed group-based trajectory modeling to chart the FBG trajectories spanning from 1996 to 2005. Employing logistic regression, we gauged the odds ratios (ORs) and 95% confidence intervals (CIs) to draw connections between initial alcohol consumption and FBG trajectory patterns, whilst controlling for predominant potential confounders.

**Results:**

Our cohort comprised 2,578 women in midlife, ranging in age from 42 to 52, each having a minimum of three subsequent FPG assessments. We discerned two distinct FBG trajectories: a low-stable pattern (*n* = 2,467) and a high-decreasing pattern (*n* = 111). Contrasted with the low-stable group, our data showcased an inverse relationship between alcohol intake and the high-decreasing FBG trajectory in the fully adjusted model 3. The most pronounced reduction was evident in the highest tertile of daily servings of alcoholic beverages (OR: 0.23, 95% CI: 0.10–0.52, *p* < 0.001), percentage of kilocalories sourced from alcoholic beverages (OR: 0.30, 95% CI: 0.16–0.58, *p* < 0.001), and daily caloric intake from alcoholic beverages (OR: 0.31, 95% CI: 0.16–0.62, *p* < 0.001).

**Conclusion:**

Moderate alcohol consumption may protect against high FPG trajectories in middle-aged women in a dose–response manner. Further researches are needed to investigate this causality in midlife women.

## 1 Introduction

Diabetes stands as one of the world’s predominant metabolic maladies. Drawing from the empirical records of the International Diabetes Federation, the global incidence of diabetes among adults is gauged at 10.5%, with an anticipated escalation to 12.2% by 2045 (1). When inadequately managed, diabetes paves the way for grave sequelae including nephropathy, neuropathy, and retinopathy (2). The 2019 Global Burden of Diseases underscored that mortalities and Disability-Adjusted Life Years (DALYs) ascribed to elevated fasting plasma glucose (FPG) tallied at 6.5 million and 172.1 million, respectively (3).

The pathophysiological mechanisms of diabetes are multifactorial, encompassing genetic and environmental factors (2, 4). Epidemiological studies suggests that diabetes can be prevented with lifestyle modifications, such as adherence to a high-quality diet, increasing physical activity, and abstention from tobacco (5, 6). Relative to the aforementioned lifestyles, the impact of alcohol consumption on diabetes remains contentious. In the nuanced “Atherosclerosis Risk in Communities” analysis spearheaded by He and colleagues, a counteractive correlation was identified between alcohol intake and the peril of diabetes (7). An intricate dose–response meta-analysis, encompassing 26 forward-looking studies, delineated that mild to moderate alcoholic indulgence correlated with a diminished susceptibility to type 2 diabetes (T2D). Contrarily, pronounced alcohol consumption bore no discernible connection to such risk (8). Delving deeper through a Mendelian randomization meta-analysis, the rs1229984 variant within the alcohol dehydrogenase 1B gene was employed as a proxy for alcohol consumption. Intriguingly, no causal nexus was discerned between the rs1229984 A-allele and both diabetes incidence and glucose concentrations (9). Furthermore, Beulens and associates unearthed that moderate alcohol ingestion inversely corresponded with T2D exclusively in women (10).

Seldom researches account for the effect of alcohol consumption on longitudinally dynamic changes of FPG. Monitoring trajectory patterns of PFG over time under varying levels of alcohol exposure might offer insights into the influence of alcohol on the natural progression of FPG. Therefore, we employed group-based trajectory modeling (GBTM) to pinpoint potential FPG trajectories and investigate the correlation between alcohol consumption and FPG fluctuations over time in a substantial representative cohort of US midlife women. We postulated that there would be distinct FPG trajectories associated with alcohol consumption.

## 2 Materials and methods

### 2.1 Study population

**Figure 1:**
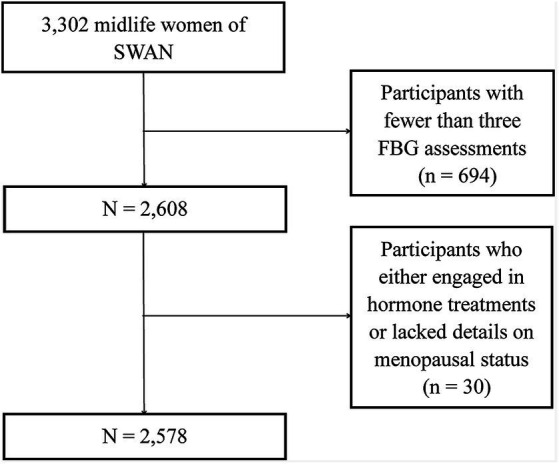
Flow chart of the study participants.

We procured data from the publicly accessible SWAN, a distinguished multi-center, longitudinal study centered on American women transitioning through midlife (11). The enlistment process, commencing in January 1996, sought out 3,302 women aged between 42 and 52, culminating their foundational visit by December 1997. Eligibility criteria mandated that these women retain a uterus and at least one unaltered ovary, have experienced a menstrual cycle within the preceding trimester, and not have partaken in hormone therapies during the same period. After their initial assessment, these women underwent annual evaluations. The SWAN’s methodology received endorsement from the pertinent institutional review boards, ensuring each participant’s informed consent. SWAN meticulously cataloged a digital compendium encompassing health profiles, physiological metrics, symptomatic reports, lifestyle nuances, and sex hormone concentrations. From this array, we dismissed participants with fewer than three FBG assessments (*n* = 694) and those who either engaged in hormone treatments or lacked details on menopausal status (*n* = 30). This rigorous selection process resulted in a refined cohort of 2,578 individuals for subsequent examination (Figure 1).

### 2.2 Alcohol consumption assessment

Alcohol consumption was evaluated using a questionnaire that encompassed the daily number of alcoholic beverage servings, daily dietary kilocalorie estimates from such beverages, and the percentage of kilocalories derived from them. Daily consumption was ascertained through a modified 1995 Block Food Frequency Questionnaire (12, 13). Dietary intake computations relied on data provided by the United States Department of Agriculture (14).

### 2.3 Fasting blood glucose assessment

FBG was assayed from serum samples at each follow-up visit. The FBG level was determined by hexokinase method.

### 2.4 Assessment of other variables

The SWAN provide data on age, race/ethnicity, household income, body mass index, physical activity (15), smoking, menopausal status, estradiol, dehydroepiandrosterone sulfate, follicle-stimulating hormone, sex hormone-binding globulin, testosterone, thyroid-stimulating hormone, and hypertension.

### 2.5 Statistical analysis

GBTM was utilized to identify potential FBG trajectories from visit 0 (1996–1997) to visit 7 (2003–2005). After determining the number and shape of the trajectories, general characteristics between different FBG trajectory patterns were evaluated using the Mann–Whitney test and the chi-square test. Multiple logistic regression models were constructed to estimate ORs with 95% CIs for FBG trajectories at various alcohol consumption levels. Tests for linear trends were executed using alcohol consumption as continuous variables within the logistic regression models. Potential interactions between alcohol consumption and other covariates were examined by constructing regression models that included main effects and their interaction terms. Sensitivity analyses were conducted using FBG from visit 1 to visit 7, as opposed to from visit 0 to visit 7. Analyses were executed using Stata version 15.1. All presented *p*-values are two-sided.

## 3 Results

### 3.1 Trajectories of fasting blood glucose

**Figure 2:**
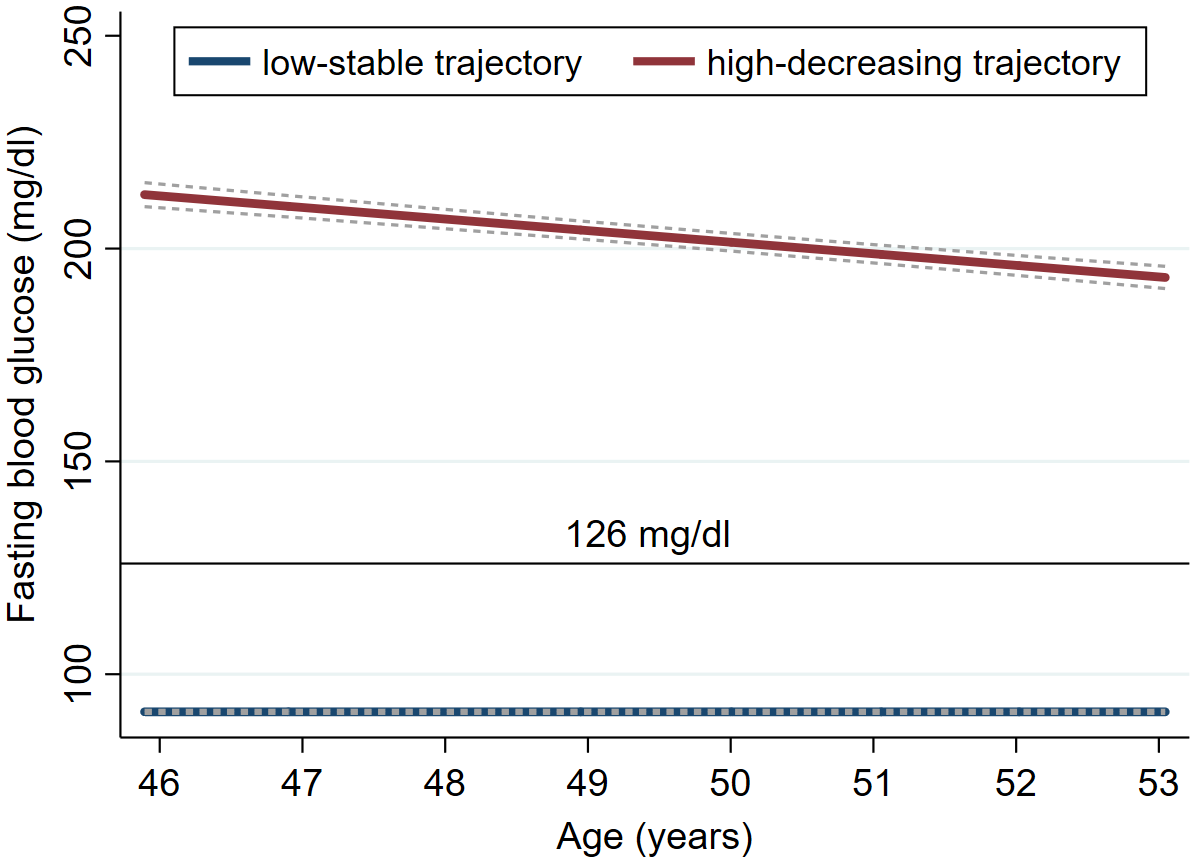
Fasting blood glucose trajectory patterns with 95% confidence intervals in the SWAN. The blue line is the low-stable trajectory and the red line is the high-decreasing trajectory.

Two distinct FBG trajectory groups emerged from visit 0 to visit 7 among midlife women (Figure 2). Group 1 (95.69%, *n* = 2,467) maintained consistently low FBG levels, averaging between 92.78 mg/dL at visit 0 and 90.08 mg/dL at visit 7, termed the “low-stable pattern.” Group 2 (4.31%, *n* = 111) began with elevated FBG levels, which subsequently declined, ranging from 204.31 mg/dL at visit 0–179.69 mg/dL at visit 7, termed the “high-decreasing pattern.”

### 3.2 Baseline characteristics of participants

Table 1 presents the demographic characteristics of midlife women across different FBG trajectory groups. Compared to those in the low-stable FBG group, women in the high-decreasing FBG group were predominantly Black/African American, early perimenopausal, had lower family income, engaged in less physical activity, consumed less alcohol, and had decreased serum levels of dehydroepiandrosterone sulfate and sex hormone-binding globulin. However, they exhibited a higher BMI and elevated serum testosterone levels.

**Table 1:** Baseline characteristics of participants according to FBG trajectory patterns.

Variables	Total participants	FBG trajectory patterns		*P*
		Low- stable	High- decreasing	
N (%)	2,578	2,467 (95.69)	111 (4.31)	
Age (years), median (IQR)	46 (44–48)	46 (44–48)	46 (44–48)	0.774
Race, %				<0.001
Black/African American	718 (27.85)	672 (27.24)	46 (41.44)	
Chinese/Chinese American	231 (8.96)	229 (9.28)	2 (1.80)	
Japanese/Japanese American	251 (9.74)	249 (10.09)	2 (1.80)	
Caucasian/White Non-Hispanic	1,239 (48.06)	1,190 (48.24)	49 (44.14)	
Hispanic	139 (5.39)	127 (5.15)	12 (10.81)	
Annual household income, %				<0.001
<50,000$	1,165 (46.45)	1,093 (45.52)	72 (67.29)	
≥50,000$	1,343 (53.55)	1,308 (54.48)	35 (32.71)	
Body mass index, %				<0.001
<25 kg/m^2^	1,241 (49.92)	1,232 (51.76)	9 (8.49)	
≥25 kg/m^2^	1,245 (50.08)	1,148 (48.24)	97 (91.51)	
Physical activity score, mean (SD)	6.66 ± 1.30	6.68 ± 1.30	6.15 ± 1.23	<0.001
Smoker, %				0.339
No	2,166 (85.14)	2075 (85.29)	91 (81.98)	
Yes	378 (14.86)	358 (14.71)	20 (18.02)	
Hypertension, %				<0.001
No	2048 (79.84)	1989 (81.05)	59 (53.15)	
Yes	517 (20.16)	465 (18.95)	52 (46.85)	
Menopausal Status, %				0.035
Early perimenopausal	1,166 (45.23)	1,105 (44.79)	61 (54.95)	
Premenopausal	1,412 (54.77)	1,362 (55.21)	50 (45.05)	
Estradiol (pg/mL), median (IQR)	55.35 (33.23–87.95)	56.1 (33.5–88.2)	46.1 (31.8–78.15)	0.084
Dehydroepiandrosterone sulfate (μg/dL), median (IQR)	115.6 (76.3–171.1)	117.29 (77.4–172.05)	83.4 (52.9–141)	<0.001
Follicle-stimulating hormone (mIU/mL), median (IQR)	16 (11–26.4)	16.1 (11–26.4)	14.5 (10.2–25.5)	0.154
Sex hormone-binding globulin (nM), median (IQR)	41 (27.9–57.6)	41.6 (28.5–58.24)	27 (17.8–37.3)	<0.001
Testosterone (ng/dL), median (IQR)	41.2 (29.8–55.8)	41.1 (29.7–55.5)	48.4 (32.99–64.2)	0.008
Thyroid stimulating hormone (mIU/mL), median (IQR)	1.91 (1.34–2.84)	1.9 (1.34–2.85)	1.99 (1.5–2.76)	0.343
Daily number of servings of alcoholic beverages, mean (SD)	0.24 (0.51)	0.25 (0.52)	0.06 (0.15)	<0.001
Percent kilocalories from alcoholic beverages, mean (SD)	2.41 (4.70)	2.49 (4.77)	0.66 (1.98)	<0.001
Daily dietary kilocalories from alcoholic beverages (kcal), mean (SD)	43.09 (90.55)	44.49 (92.06)	11.19 (30.5)	<0.001

Chi-square test for categorical variables; Mann–Whitney test or *T*-test for continuous variables. FBG, fasting blood glucose; IQR, inter-quartile range; SD, standard deviation.

### 3.3 Association between alcohol consumption and FBG trajectory patterns

Of the participants, 2,460 provided complete data regarding alcohol consumption. Table 2 displays the relationships between alcohol consumption and FBG trajectory patterns. We observed an inverse correlation between alcohol consumption and the high-decreasing FBG pattern in models adjusted for age and race. This association was most pronounced in the highest tertile for various alcohol consumption metrics, including daily servings (OR: 0.17, 95% CI: 0.08–0.36, *p* < 0.001), percent kilocalories (OR: 0.26, 95% CI: 0.15–0.46, *p* < 0.001), and daily kilocalories (OR: 0.26, 95% CI: 0.15–0.46, *p* < 0.001). Upon adjusting for factors such as annual household income, BMI, physical activity, smoking, and hypertension status, the ORs for the high-decreasing FBG trajectory pattern were as follows: 0.17 (95% CI: 0.08–0.36, *p* < 0.001) in the highest tertile for daily servings of alcoholic beverages, 0.29 (95% CI: 0.15–0.55, *p* < 0.001) for percent kilocalories from alcohol, and 0.31 (95% CI: 0.16–0.57, *p* < 0.001) for daily dietary kilocalories from alcohol. This correlation remained robust even after additional adjustments, including hormonal factors, and was most pronounced in the uppermost tertiles of alcohol consumption. Sensitivity analyses, which excluded initial FBG readings, upheld these relationships with minor deviations. No interactions between alcohol consumption and other variables were identified in the third model (data not shown).

**Table 2:** Odds ratios for the association between alcohol consumption and FBG trajectory patterns.

	*N*	Cut-off	Model 1	*P*	Model 2	*P*	Model 3	*P*
Daily number of servings of alcoholic beverages								
T1	1,224	0	1.0 (reference)		1.0 (reference)		1.0 (reference)	
T2	565	0.1	0.53 (0.32–0.87)	0.013	0.57 (0.33–0.99)	0.046	0.50 (0.28–0.90)	0.019
T3	671	≥0.2	0.17 (0.08–0.36)	<0.001	0.17 (0.08–0.36)	<0.001	0.23 (0.10–0.52)	<0.001
P for trend				<0.001		<0.001		<0.001
Percent kilocalories from alcoholic beverages, %								
T1	1,224	0	1.0 (reference)		1.0 (reference)		1.0 (reference)	
T2	417	0.15–1.38	0.49 (0.27–0.89)	0.018	0.56 (0.30–1.04)	0.069	0.49 (0.25–0.95)	0.035
T3	819	≥1.39	0.26 (0.15–0.46)	<0.001	0.29 (0.15–0.55)	<0.001	0.30 (0.16–0.58)	<0.001
P for trend				<0.001		<0.001		<0.001
Daily dietary kilocalories from alcoholic beverages, kcal								
T1	1,224	0	1.0 (reference)		1.0 (reference)		1.0 (reference)	
T2	416	4.12–23.15	0.50 (0.28–0.89)	0.02	0.53 (0.28–1.01)	0.054	0.45 (0.23–0.90)	0.024
T3	820	≥23.16	0.26 (0.15–0.46)	<0.001	0.31 (0.16–0.57)	<0.001	0.31 (0.16–0.62)	<0.001
P for trend				<0.001		<0.001		<0.001

Model 1 adjusted for age and race. Model 2 adjusted for covariates in model 1 and family income, body mass index, smoking, physical activity, and hypertension. Model 3 adjusted for covariates in model 2 and menopausal status, estradiol, dehydroepiandrosterone sulfate, follicle-stimulating hormone, sex hormone-binding globulin, testosterone, and thyroid stimulating hormones. FBG, fasting blood glucose.

## 4 Discussion

Existing research on the association between alcohol consumption and FBG trajectory patterns is sparse, particularly among middle-aged women undergoing menopausal transitions. Using SWAN data, we discovered that, in comparison to the low-stable FBG trajectory pattern, a higher initial alcohol consumption inversely correlated with the high-decreasing FBG trajectory pattern in 2,460 US midlife women, after controlling for potential confounders. Specifically, women in the highest tertile of daily servings of alcoholic beverages, percent kilocalories from alcohol, and daily dietary kilocalories from alcohol exhibited 77, 70, and 69% reduced odds, respectively, of exhibiting the high-decreasing FBG trajectory pattern compared to those in the lowest alcohol consumption tertile.

This previously unreported reduced risk of the high-decreasing FBG trajectory pattern associated with higher alcohol consumption in midlife women offers insights for future research into the initial effects of alcohol on FPG’s natural progression. While the underlying biological mechanisms remain largely speculative, insulin’s role appears central. In the 1988 Nurses’ Health Study, Stampfer et al. found that women consuming over 15 g/day of alcohol had a relative diabetes risk of 0.6 (95% CI: 0.3–0.9) (16). A randomized controlled trial (RCT) with postmenopausal women indicated that moderate alcohol intake (30 g/d) enhanced insulin sensitivity by 7.2% compared to non-consumption (17). Another RCT with the same demographic determined that 6 weeks of moderate alcohol consumption (25 g/d) positively influenced insulin sensitivity, adiponectin levels, and lipid profiles (18). Given alcohol’s energy content of 7.1 kcal/g (19), the median alcohol intake in our study was approximately 12 (5–23) g/d, categorizing it as moderate consumption. After excluding women with alcohol intakes exceeding 30 g/d, the inverse relationships between alcohol consumption and the high-decreasing FBG trajectory pattern remained significant with only minor variations.

The most recent meta-analysis indicated a gender-specific reduction in T2D risk, particularly among females (20). Factors unique to females, such as menopausal status and hormonal levels, could underpin this observation. Another meta-analysis revealed that both testosterone and sex hormone-binding globulin have gender-specific correlations with glycemic status and T2D risk (21). A nested case–control study focusing on postmenopausal women found that estradiol and sex hormone-binding globulin modulate the protective effect of alcohol intake (≥ 15 g/d) against T2D, resulting in a 12–21% decrease in ORs (22). Nonetheless, our findings suggest that the association between alcohol consumption and FBG trajectory patterns remains irrespective of menopausal status and ovarian hormones. Thorough epidemiological and molecular biological investigations are necessary to elucidate the intricate interactions between sex hormones and the relationship between alcohol intake and FBG trajectory patterns.

Our research boasts several merits. We presented a novel perspective on the link between alcohol consumption and FBG trajectory patterns. The inclusion of a nationwide sample of midlife women minimizes selection bias, and by assessing alcohol consumption from three angles, we mitigate measurement bias. However, there are inherent limitations. While our study derives FBG trajectory patterns from 8 years of longitudinal data, a more extended trajectory analysis remains essential for deeper insights into FPG trajectory groups, and to preempt potential reverse causation. Although we accounted for numerous confounders, the influences of unaccounted or residual confounding cannot be dismissed. Moreover, utilizing dietary recalls to gauge alcohol consumption inherently introduces recall bias. Finally, the potential change of exposure or other covariates over time may lead to bias.

## 5 Conclusion

To summarize, employing the GBTM approach, we explored the correlation between alcohol consumption and longitudinal FBG trajectories. Our findings highlight that increased initial alcohol consumption is inversely related to the high-decreasing FBG trajectory pattern in US midlife women, even after adjusting for potential confounders. These observations warrant validation in comprehensive prospective cohort studies augmented by molecular biology.

## Data availability statement

The original contributions presented in the study are included in the article/supplementary material, further inquiries can be directed to the corresponding authors.

## Ethics statement

The studies involving humans were approved by the Institutional Review Boards (University of Michigan, Massachusetts General Hospital, Rush University Medical Center, University of California, Davis, and Kaiser Permanente, University of California, Los Angeles, Albert Einstein College of Medicine, and University of Pittsburgh). The studies were conducted in accordance with the local legislation and institutional requirements. The participants provided their written informed consent to participate in this study. Written informed consent was obtained from the individual(s) for the publication of any potentially identifiable images or data included in this article.

## Author contributions

XZW: Writing – original draft, Writing – review & editing. SL: Writing – original draft, Writing – review & editing. XWW: Writing – review & editing. PG: Writing – original draft, Writing – review & editing. JC: Writing – original draft, Writing – review & editing.
